# Abundance of African invader fly, *Bactrocerainvadens* drew, tsuruta and white (diptera: tephritidae) and influence of weather parameters on trap catches in mango in the Volta region of Ghana

**DOI:** 10.1186/s40064-016-2644-0

**Published:** 2016-07-04

**Authors:** Charles Amankwa Adzim, Maxwell Kelvin Billah, Kwame Afreh-Nuamah

**Affiliations:** African Regional Postgraduate Programme in Insect Science, University of Ghana, Accra, Ghana; Department of Animal Biology and Conservation Sciences, University of Ghana, Accra, Ghana; Forest and Horticultural Crop Research Centre, University of Ghana, Kade, Ghana

**Keywords:** *Bactrocerainvadens*, Agro ecological zones, Correlation, Rainfall, Temperature

## Abstract

The seasonal abundance of African Invader fly, *Bactrocerainvadens* and the influence of temperature and rainfall on fly catches was determined in two agro ecological zones; moist semi-deciduous forest area and the coastal grassland area of the Volta Region of Ghana for year of mango production. Traps containing methyl eugenol were used in monitoring the abundance of the Africa invader fly, *Bactrocera invadens* where data on both temperature and rainfall were collected from Meteorological Services of Ghana in Volta region. A total of 49,322 organisms captured, 45,829 were identified as *Bactrocerainvadens* and 3493 were non-fruit fly. There were significant differences (p < 0.05) in the number of *Bactrocerainvadens* captured between the agro ecological zones with relative fly densities of 5.06 F/T/D in moist semi deciduous forest area and 2.38 F/T/D in the coastal grassland zone. The result shows that climatic factors affected *Bactrocerainvadens* differently in different agro ecological area. There was negative correlation and highly significant (p < 0.001) correlation between fruit flies and temperature whereas there was negative correlation and high significant (p < 0.01) difference between rainfall in the moist semi deciduous forest area. In the coastal grassland area, there was negative correlation and highly significant (p < 0.001) between *Bactrocerainvadens* for both rainfall and temperature. *Bactrocerainvadens* activities peaked differently during the study period due to favourable climatic conditions. The activities of *Bactrocerainvadens* peaked during weeks 7 and 29 in the moist semi deciduous forest area while their activities peaked during weeks 3 and 24 for the coastal grassland areas. Both agro ecological zones recorded the presence of *Bactrocerainvadens*, their number and proportion varied considerably with associated effects of the weather parameters on their abundance. The effect of weather parameters on the abundance of bactrocera invadens requires the development of degree day models to manage them.

## Background

Mango continues to be an important tropical fruit for Sub-Saharan African economies, serving as a major source of nutrition for the rural population, playing a role in reducing poverty and a potential export product (Vayssieres et al. 2008). However, fruit fly pests cause economic damage in terms of direct produce losses, as well as interception, seizure, and banning of infested fruits destined for export (Ekesi and Billah 2006; Vayssières et al. 2013). The Africa Invader fly, *Bactrocerainvadens*, the most devastating of the Sub-Saharan fruit fly pests, was first detected in Africa in 2003(Lux et al. 2003; Drew et al. 2005) and first detected in Ghana in 2005 (Billah et al. 2006). After its detections in Ghana, the flies have almost completely replaced existing species of fruit flies spreading in over thirty (30) African countries (De Meyer et al. 2011) and causing direct damage ranging from 30 to 80 % of the crop depending on the cultivar, locality and season (Ekesi et al. 2006; Rwomushana et al. 2008; Vayssieres et al. 2009). Weather factors such as relative humidity, rainfall, and temperature and wind velocity have great influence on fruit fly populations. For instance, outside the optimum temperature range of approximately 18–27 °C mortality increases and there are upper and lower lethal thresholds beyond which no individuals survive long enough to complete development (Fletcher 1987). According to Rwomushana et al. (2008) the *B*. *invadens* survives well in moist weather and high temperatures.

In the West Africa sub region, mango production increased from 15,000 to over 22,000 tonnes a rise of about 7000 tonnes in 2012 to the European market (ECOWAS-TEN 2012). However, the mangoes were intercepted, confiscated and destroyed in the European harbours because of this quarantine insect, thus leading to economic losses to the exporters and the whole horticultural sector (ACP-EU Newsletter 2013). For instance, there were over 90 interception of mango from the sub-region the highest from Cote d’lvoire leading with 34 interceptions followed by Ghana (28) valued at 2.8 million Pounds (FCFA 1.83 billion) at a rate of 30,000 Pounds per interception in the year 2012 (ECOWAS-TEN, 2012). In light of the numerous fruit interceptions to the major markets, especially the European Union, the European Commission initiated a scoping study across the West African Sub-region to access the levels of the fruit fly damage and to partner with countries of the sub-region in combating the menace with a concerted effort. Based on the outcome and recommendations of the study, National Fruit Fly Committees were formed in the partner countries with the overall mandate or function of coordinating all the fruit fly activities in each country to ensure a systematic and harmonized way of managing the pest. The stakeholder awareness creation seems not to yield the needed results because there is relative abundance of *B*. *invadens* overshadowing other species still affecting the industry after tens (10) years of its invasion in Ghana (PPRSD-MOFA 2010; COLEACP 2010). There is therefore the need to study weather factors on the abundance of fruit flies across so that we can monitor them based on the changes in weather.

The study was to monitor the abundance of the Africa invader fly, *Bactrocera invadens* by using traps and as well determine the effects of temperature and rainfall on trap catches in two separate agro ecological zones of the Volta region of Ghana.

## Methods

### Study site and experimental design

**Fig. 1:**
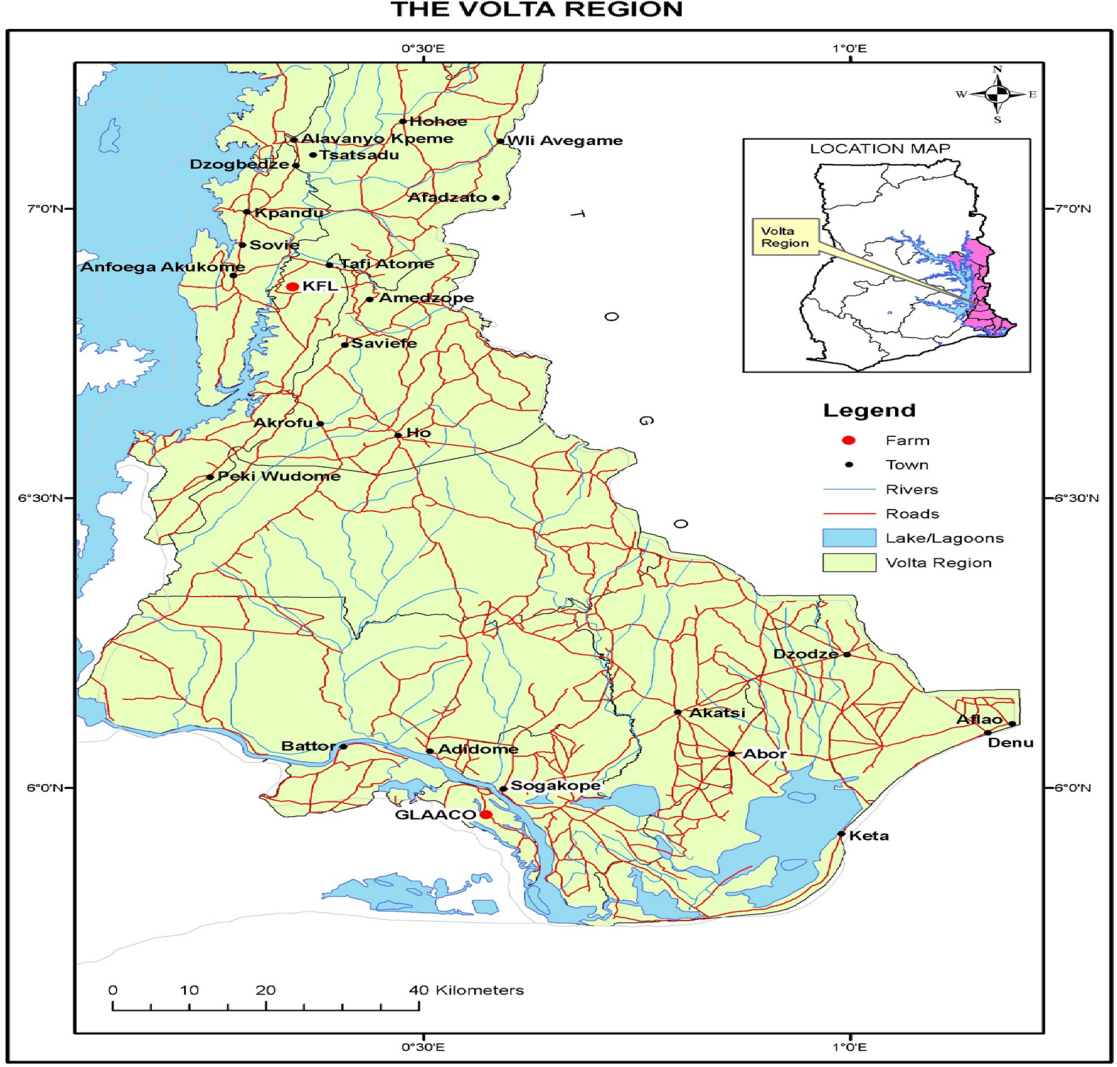
Map of Volta Region showing the study site

The study was conducted in two agro ecological zones; moist semi-deciduous forest and coastal grassland zone in the Volta region of Ghana for forty-three weeks (43). Two mango producing farms were selected in each zones; Volta Integrated Agricultural Development- Kingdom fruits, Tafi in the moist semi-deciduous zone and the Ghana Libyan Arab Agricultural Company-(GLAACO), Morkordzi farms located in the coastal grassland zone as shown in the Fig. 1. The study was conducted in one hectare plot each of two mango varieties; Kent and Keitt with planting distances between trees of 10 × 10 m. In each plot, the first tree was selected and tagged, and then tenth tree was also counted tagged as second tree. The third and four were also selected after a count of ten trees in a square form in the hectare plot. The fifth tree was selected at the centre of the four trees in the field.

Rainfall and temperature data were collected from the Ghana Meteorological Services department at the regional office in the Volta region.

### Preparation and setting of traps

**Fig. 2:**
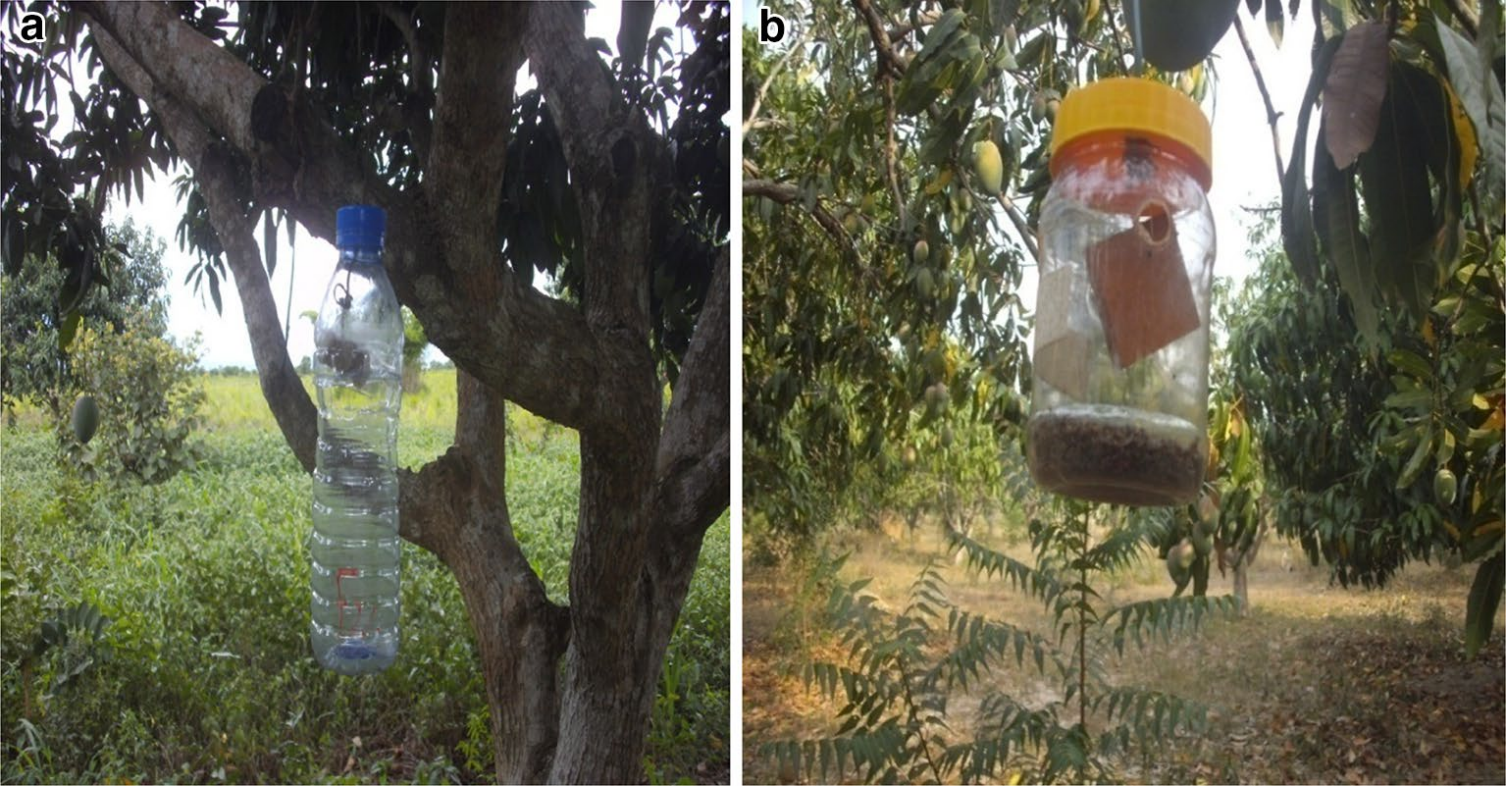
Trap types used in the study. **a** Mineral water bottle trap, **b** *yellow*-top trap

Two different homemade traps the mineral water bottle (MWB) trap (Fig. 2a) and the Yellow-Top Plastic (YTP) trap (Fig. 2b), with compacted fiber wood block, soaked in methyl eugenol marketed as “Stop Mating Block” (Stonehouse et al. 2002) were hanged on the branches of the mango trees. In each plot of the mango orchard, five (5) 500-ml mineral water bottles were deployed. Two windows 3 × 2 cm were cut on opposite sides of the bottles at 7 cm from the top, and the base perforated to allow water drainage. The lid of the trap was perforated and a binding wire (diameter 0.1 cm) with a knot at 7 cm from the hook at the tip and was passed through to prevent slipping. A cotton wick with a knot was soaked in a mixture of liquid Methyl Eugenol (ME) and an insecticide (Deltamethrin) in a ratio of 4:1, and placed in the hook at the tip of the wire.

The Yellow-Top plastic trap was locally made (Fig. 2b). Four circular holes were perforated at the upper part of the plastic container to serve as entry point for the flies. Imported polymeric plugs or fiber wood blocks (Stop Mating Block) contains methyl eugenol and killing agent (Deltamethrin) was hanged at 7 cm in small plastic holder and placed in the container. A nylon thread was placed through the yellow-top plastic and hanged on the tree.

In each farm ten traps per plot (5 MWB + 5 YTP) were hanged at heights of 1.5–4 m above ground (depending on tree age and canopy architecture) at a distance of 50 m apart to avoid interference with each other (Ekesi and Billah 2007). The traps were placed in an alternating fashion in semi-shaded spots in the upwind part of the canopy such that the branches and leaves were close (but not touching the traps) to serve as landing places for the flies before entering the traps (Ekesi and Billah 2007). Solid Grease was smeared at middle third of the hanging wire or trap rope to prevent ants from preying on the catches. The ME in the Mineral Water bottle trap was replaced every four weeks and the Yellow-Top trap was replaced every six weeks. The fruit fly catches from the traps were collected at weekly intervals, counted, and the counts from the two varieties and traps were put together for each farm into plastic collection vials containing 70 % alcohol for preservation and identification at the African Regional Postgraduate Programme in Insect Science (ARPPIS) Laboratory. The catches from the two farms were compared and also effects of weather parameters analyzed.

### Identification of fruit flies

Taxonomic keys by White and Elson-Harris (1992), De Meyer (1996, 1998, 2000), Billah et al. (2007), and were used with aid of a Lecia EZ4 D microscope to identify the various species of fruit flies collected from the traps. The identification was done in the African Postgraduate Programme in Insect Science (ARPPIS) Laboratory. Confirmation and inability to identify species were referred to M.K. Billah, a Fruit Fly Taxonomist in the Department of Animal Biology and Conservation Science, University of Ghana, Legon.

### Data analysis

All the flies captured were counted and their relative fly densities calculated based on IAEA (2003) specification to determine the average number of flies captured in one trap in a day that the trap was exposed to the field.

Relative fly density = total number of flies (F)/total number of traps (T)/average number of days (D). relative density = F/T/D. The percentage trap catches were also determined for the catches for the two locations and varieties as well. The data was analyzed using the R-Statistical software version 3.2.3 and linear regression model was used to analyze the correlation between weather parameters.

## Results

### Population trends of fruit flies

**Fig. 3:**
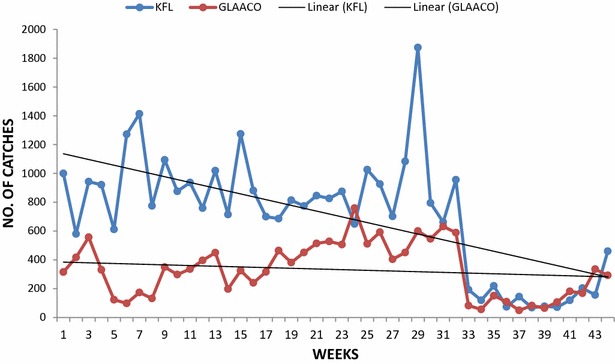
Weekly trap catches of fruit flies from moist semi deciduous forest area and coastal grassland area

**Fig. 4:**
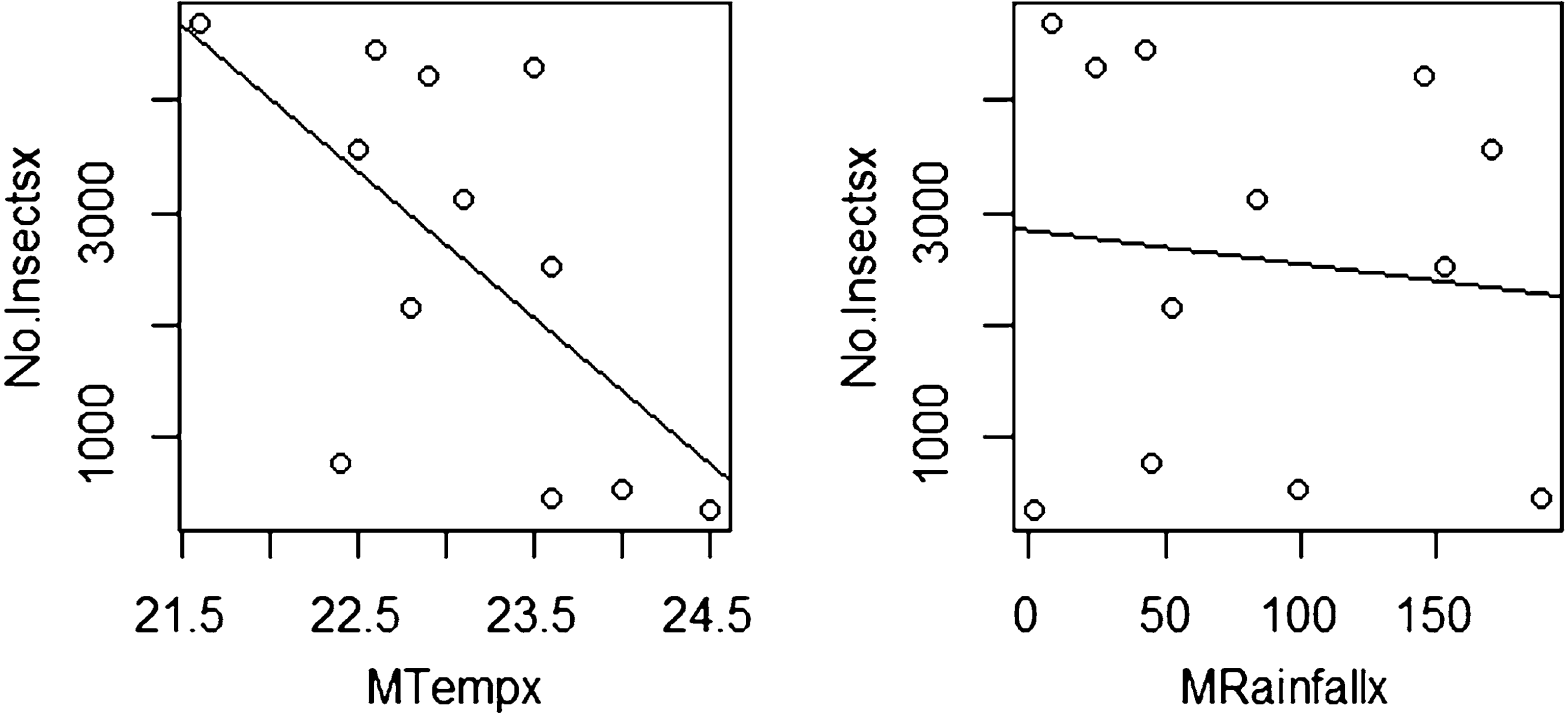
Abundance of *Bactrocera* *invadens* and climatic factors during the study in the moist semi deciduous forest area

**Fig. 5:**
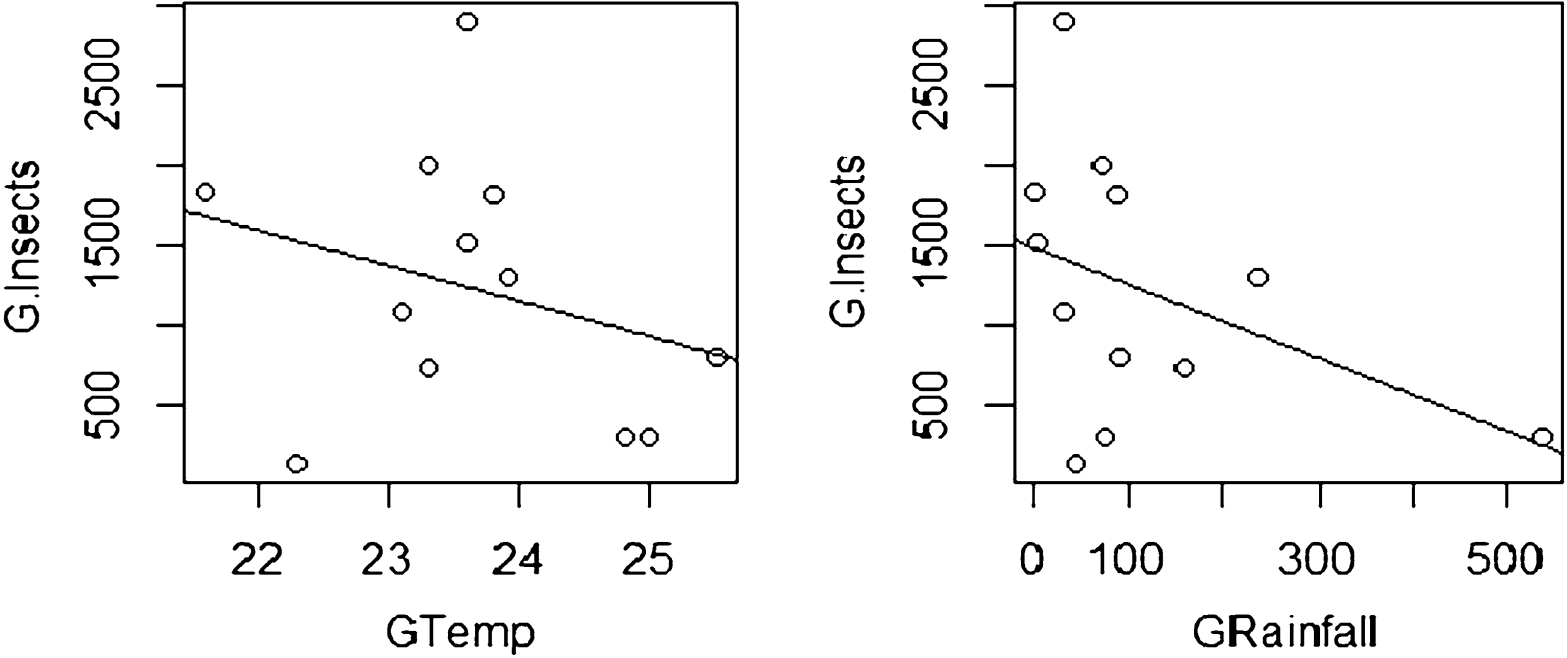
Abundance of *Bactrocera* *invadens* and climatic factors during the study in the coastal grassland area

A total of 45,829 fruit flies were captured in the two agro ecological zones. All fruit flies captured were identified as *Bactrocerainvadens* (Table 1). Moist semi-deciduous zone recorded 31,171 representing 68.02 % whilst the coastal grassland recorded a total of 14,658 (31.98 %) (Table 1). A relative fly density of 5.06 F/T/D was observed at the moist semi deciduous forest whilst coastal grassland recorded 2.38 F/T/D. The high relative fly density in the moist semi deciduous forest area shows that there is high incidence of the bactrocera invadens there compared to the coastal grassland area (Figs. 3, 4, 5).

**Table 1:** Trap captures, percentages, and relative fly density of fruit flies in trees of different varieties at different locations (No. of traps was 10 each, and days of exposure were 308)

Location	Variety	No. of flies (F)	%	Relative fly density F/T/D
KPFL	Kent	14,241	31.07	4.62
	Keitt	16,930	36.94	5.50
GLAACO	Kent	7096	15.48	2.30
	Keitt	7562	16.50	2.46
Total		45,829	100

During the research, *Bactrocerainvadens* populations kept fluctuation throughout the study periods across the two agro ecological zones. The moist semi deciduous forest area recorded the highest numbers in the first four weeks than in the coastal grassland area. It was during this period that the target substance mango fruits where reducing in numbers. The corresponding temperature and rainfall for the period were 23.6 °C, 23.9 °C and 152.9 mm and 235.6 respectively. *Bactrocerainvadens* populations fluctuated across the two ecological zones during the study period. The moist semi deciduous forest area recorded higher trap catches than the coastal grassland area over the first four weeks, during which the numbers of mango fruits were decreasing. The captures peaked when the most fruits were available for fruit flies, at the 29th week in the moist semi deciduous forest area and the 24th week in the coastal grassland. There was a sharp drop in catches between weeks 33 and 43, corresponding to the period of flowering and fruiting. In addition, rainfall decreased and temperature increased in both zones, which may have contributed to the reduced catches. Overall, the catches were higher in the moist semi deciduous area than in the coastal grassland area. Also, the mean temperature and rainfall were higher in the moist semi deciduous forest than the coastal grassland.

### Correlation between temperature, rainfall and fruit fly catches

The regression coefficient for the relationship between trap capture and temperature was negative and the difference of slope from zero baseline was extremely highly significant (p < 0.001) for both the moist semi deciduous forest and the coastal grassland zones (Table 2). In addition, the regression coefficient for the relationship between trap capture and mean rainfall was negative. The difference of slope from zero baseline was extremely highly significant for the coastal grassland and highly significant for the moist semi deciduous forest.

**Table 2:** Correlation between climatic factors and Bactrocera invadens abundance in the two agro ecological zones

Climatic factors/zones	Moist semi deciduous forest		Coastal grassland	
	Regression coefficient P	Regression coefficient P	Regression coefficient P	Regression coefficient P
Mean temperature (°C)	−0.6027	2.2e−16^***^	−0.2893	2.2e−16***
Mean rainfall (mm)	−0.1159	0.0010^**^	−0.4134	2.2e−16***

** High significant, *** Highly significant

## Discussion

After a year of weekly monitoring of the abundance of *Bactrocerainvadens* using trap and comparing the effects of temperature and rainfall on trap catches, a total 45,829 (92.92 %) fruit flies and 3493 (7.08 %) non-fruit fly organisms were identified. It was no doubt that all the flies captured were identified as the Africa Invader fly, *Bactrocerainvadens* confirming earlier works that the flies are known to be greatly attracted to methyl eugenol (ME) which has both olfactory as well as phagostimulatory action and can attract fruit flies from a distance of 800 m, indicating its effectiveness in managing *Bactrocerainvadens*. (White and Elson-Harris 1992; Roomi et al. 1993, Billah et al. 2006). The high numbers of *Bactrocerainvadens* confirms the findings by Lux et al. (2003), Billah et al. (2006), Mwatawala et al. (2009) and Nboyine et al. (2012), that the flies has dominated other species of fruit flies since its introduction to Africa in 2003, and Ghana in 2005. After monitoring the flies on weekly basis, the moist semi-deciduous forest area recorded high relative abundance density of 5.06 F/T/D than the coastal grassland with 2.38 F/T/D. The relative abundance was in the range stated by Nboyine et al. (2012) where the coastal grassland area recorded fly density of the range 0.02–22.25 and 0.08–121.39 flies per trap per day and in the moist semi-deciduous zone of range 0.02–31.40 and 0.01–104.23 in the major and minor seasons.

The analysis showed that in the moist semi deciduous forest area, both climatic factors showed a negative correlation with *Bactrocerainvadens* abundance. The mean temperature showed extremely high significant correlation while the mean rainfall showed high significant correlation coupled with population fluctuation during the study period in the area. Peaked activity of *Bactrocerainvadens* was observed in the weeks of the months of August and November. It was observed that despite the higher numbers both rainfall and temperature reading were low indicating that they were optimal for the abundance of *Bactrocerainvadens*. Confirming Fletcher (1987) that temperatures outside the optimum temperature range of approximately 18–27 °C increases mortality beyond which no individuals survive long enough to complete development. In the weeks of the month of September and October when fruits started to set after flowering and also in last week in August saw a drop in the population of flies for both farms. However, the population of flies increased in the late weeks of October through to early December when the fruits were matured for harvesting.

According to Rwomushana et al. (2008) the *B*. *invadens* strives well in moist weather and high temperatures hence the high numbers of *B*. *invadens* during weeks in most of months during high rainfall pattern. Heavy rainfall led to high vegetative growth of grasses and shrubs in the farms making difficult for the farmers to control weeds. In the moist semi deciduous forest because of the high rainfall pattern the farm was very weedy making it tedious to collect dropped fruits for burial on time, serving as medium for the larvae to pupate in the soil for continuation of the life cycle. The rainfall therefore had influence on the numbers of *Bactrocerainvadens* in this area. The coastal grassland area on the other hand it was observed that there was low vegetation. The farmer as a good management strategies used cattle, sheep and goats to graze in the farms where the animals ate fallen fruits thus breaking the life cycle of the *Bactrocerainvadens* thus contributing to low numbers.

For instance, in September a high rainfall recorded led to flower and fruit drops but the *B*. *invadens* catches were still high. The influence of rainfall and temperature on *B*. *invadens* population does not confirm other researches that fruit fly catches had positive correlation with temperature and rainfall rather a negative correlation (Sarada et al. 2001). Other authors confirmed that there is positive correlation between temperature and negative correlation with rainfall (Bagle and Prasad 1983, Sushilkumar et al. 1997). A degree day model could be a predictive tool of fruit fly abundance in an area to assist the farmer in effective management of fruit flies. Continuous trapping and monitoring of fly populations no matter the state of the fruit in the farm, is the best way to manage the population of *Bactrocerainvadens* (ACP-EU Newsletter 2013).

## Conclusion

Temperature and rainfall have great influence on the growth and development of fruit flies in mango during main season and off season. Traps are used detect the presence of flies to determine areas of high infestation and to find the characteristics of the pest in the area. Continuous trapping and monitoring of fly populations no matter the state of the fruit in the farm, is the best way to manage the incidence of *Bactrocerainvadens*. The results of the data will provide information for early control measures for all stakeholders in the mango industry. The *Bactrocerainvadens* reached its peak abundance when conditions are favourable for their breeding and activities. The weather parameters affect the activities of the flies in the different agro ecological zone pointing to the differences in the numbers trapped. The trap type also had influence on the number of flies captured. The activities of *Bactrocerainvadens* peaked in the months of June, August, November and December with negative correlation between trap catches. It was evident that rainfall contributed to growth of grasses in both agro ecological which therefore calls for good sanitation and best integrated pest management approach to manage to minimize damage to mango.
